# Role of the ribosome-associated protein PY in the cold-shock response of *Escherichia coli*

**DOI:** 10.1002/mbo3.68

**Published:** 2013-02-19

**Authors:** Fabio Di Pietro, Anna Brandi, Nadire Dzeladini, Attilio Fabbretti, Thomas Carzaniga, Lolita Piersimoni, Cynthia L Pon, Anna Maria Giuliodori

**Affiliations:** 1 Laboratory of Molecular Biology and Biotechnology, School of Biosciences and Biotechnology, University of Camerino62032, Camerino (MC), Italy; 2 Dipartimento di Bioscienze, Università degli Studi di Milano20133, Milan, Italy; 3 Department of Molecular, Cellular and Developmental Biology, University of MichiganAnn Arbor, Michigan, 48109

**Keywords:** Cold shock, protein PY, translation initiation, translation regulation

## Abstract

Protein Y (PY) is an *Escherichia coli* cold-shock protein which has been proposed to be responsible for the repression of bulk protein synthesis during cold adaptation. Here, we present in vivo and in vitro data which clarify the role of PY and its mechanism of action. Deletion of *yfiA*, the gene encoding protein PY, demonstrates that this protein is dispensable for cold adaptation and is not responsible for the shutdown of bulk protein synthesis at the onset of the stress, although it is able to partially inhibit translation. In vitro assays reveal that the extent of PY inhibition changes with different mRNAs and that this inhibition is related to the capacity of PY of binding 30S subunits with a fairly strong association constant, thus stimulating the formation of 70S monomers. Furthermore, our data provide evidence that PY competes with the other ribosomal ligands for the binding to the 30S subunits. Overall these results suggest an alternative model to explain PY function during cold shock and to reconcile the inhibition caused by PY with the active translation observed for some mRNAs during cold shock.

## Introduction

A change in the environmental temperature, a physical stress that all living organisms experience, is capable of influencing the rate of many cellular reactions as well as affecting the conformation, folding, and flexibility of macromolecules. To survive an abrupt temperature downshift (e.g., from 37°C to a temperature below 20°C), *Escherichia coli* cells transiently stop growing for a period which can last one to several hours and enter an acclimation phase during which bulk protein synthesis is drastically repressed while a set of cold-shock genes is selectively and transiently expressed (Gualerzi et al. 2003, 2011; Phadtare 2004).

Induction of cold-shock gene expression during the acclimation phase is mainly regulated by two posttranscriptional mechanisms: (i) a drastic stabilization of the transcripts (Gualerzi et al. 2003) and (ii) the specific translation of the cold-shock mRNAs (translational bias) at low temperature (Goldenberg et al. 1997; Giuliodori et al. 2004). This translational bias is due to both *cis*-acting elements present in the cold-shock transcripts which render these mRNAs suitable for the translation in the cold and to *trans*-acting factors (Giuliodori et al. 2004, 2007; Giangrossi et al. 2007). Concerning the *cis*-acting elements, it has been recently demonstrated that *cspA* mRNA, the transcript of the most studied cold-shock gene of *E. coli*, acts as an intrinsic sensor of the temperature downshifts, being able to assume functionally and structurally different conformations at 37°C or in the cold (Giuliodori et al. 2010). As for the role played by the *trans*-acting factors, the increased levels of IF1 and IF3 during cold adaptation are essential to promote translation of a select group of cold-shock mRNAs and to counter the increased tendency of the ribosomal subunits to associate in the cold, thus ensuring a sufficient pool of 30S subunits for initiating translation (Giangrossi et al. 2007; Giuliodori et al. 2007).

Protein Y (PY), known also as RaiA or YfiA (Maki et al. 2000; Agafonov et al. 2001), is one of the least functionally characterized of the cold-shock *trans*-acting factors. PY appears in the ribosomal fraction in the first hour of cold shock, remains ribosome associated throughout the cold acclimation phase with a PY/ribosome ratio between 1:10 and 1:3, and is released when growth resumes (Agafonov et al. 2001). Interestingly, PY associates with the ribosomes also during stationary phase at 37°C (Maki et al. 2000; Agafonov et al. 2001).

PY binds to the 30S subunit and to the 70S monomer at the subunit interface with the 50S subunit (Agafonov et al. 1999). The X-ray diffraction structure of the PY-ribosome crystal indicates that PY binds the region overlapping the A and P binding sites of the tRNA, in close proximity to helix 69 in the 23S rRNA, which is one of the regions responsible for subunit association (Vila-Sanjurjo et al. 2004; Polikanov et al. 2012). In agreement with this finding, it has been demonstrated that PY inhibits translation, stabilizes the 70S ribosome against dissociation, and inhibits the binding of the initiator tRNA to the 70S ribosome at low temperature (Agafonov et al. 1999; Vila-Sanjurjo et al. 2004).

PY is conserved in several bacterial species and its functional homologs are present also in the plastid of many plants (Agafonov et al. 1999; Sharma et al. 2010). Nevertheless, PY is the least conserved and least abundant of the ribosome-associated proteins and it can be considered as an auxiliary factor expressed only to overcome stress conditions (Agafonov et al. 1999). In fact, it has been postulated that this protein could act as a “storing” factor, sequestering a fraction of the ribosomes as idle 70S monomers when a lower number of translating ribosome is required by the cell (Maki et al. 2000; Sharma et al. 2010). Recently, it has been proposed that ribosome-recycling factor (RRF) and elongation factor G (EF-G) may be responsible for the PY release from the stabilized 70S monomers and for the subsequent dissociation of the 70S ribosome to 30S and 50S subunits (Sharma et al. 2010), which are then prevented from reassociation by the concerted activity of IF1 and IF3 (Giangrossi et al. 2007; Giuliodori et al. 2007).

The discovery of PY was greeted with enthusiasm by the scientific community which postulated that this protein might be the factor that modulates ribosome activity as a function of cell stress and transiently represses the synthesis of non–cold-shock proteins during cold adaptation (Vila-Sanjurjo et al. 2004; Wilson and Nierhaus 2004). However, experimental evidence in support of this hypothesis is lacking. Another open question concerns the mechanism by which the synthesis of cold-shock proteins can occur in spite of translation inhibition by PY. Furthermore, although the PY-induced inhibition of 30S initiation complex formation has been hypothesized (Vila-Sanjurjo et al. 2004), this activity has never been demonstrated.

Thus, aim of this study is to clarify the role of protein PY during cold shock and to unravel the molecular basis of the PY-dependent translation inhibition. To establish whether PY is the factor that inhibits the synthesis of non–cold-shock proteins at low temperature, we inactivated the *yfiA* gene in the *E. coli* strain MRE600 and compared the behavior of both wt and mutant strains at 37°C and during cold shock. Moreover, we compared the patterns of proteins synthesized in the two strains before and after cold shock by pulse-chase experiments. The results obtained indicate that PY is dispensable for cell growth at both 37°C and low temperature and that this protein is not responsible for turning off the synthesis of the non–cold-shock protein during the cold adaptation phase. However, PY seems to be able to reduce the translation of at least some mRNAs. To verify this finding, we performed in vitro translational experiments in the presence of increasing amounts of PY, using a set of cold-shock and non–cold-shock mRNAs and analyzed the effect of PY on 30S and 70S initiation complex formation using various molecular approaches. The present results establish that PY inhibits translation to different extent depending upon temperature and mRNA. This activity is due to the capacity of this protein to bind the 30S subunits, thus interfering with the formation of 30S initiation complexes and simultaneously favoring the association of the ribosomal subunits to form idle 70S ribosomes.

## Experimental Procedures

### General preparation

*Escherichia coli* MRE600 70S ribosomes; S100 postribosomal supernatant; S30 crude extracts; 30S and 50S ribosomal subunits; and purified initiation factors IF1, IF2, and IF3 were prepared as described previously (Giuliodori et al. 2004, 2007). tRNAfMet was kindly provided by S. V. Kirillov (Gatchina, Russia) and f[^35^S]Met-tRNA was prepared and purified as described (Rodnina et al. 1994; Brandi et al. 2004).

### Strains

*Escherichia coli* MRE600 (Cammack and Wade 1965; laboratory stock) was used to construct the deletion mutant MRE600 *ΔyfiA*; *E. coli* JM109 was used for plasmid amplification (Sambrook and Russell 2001); *E. coli* BL21 (DE3)-pLYS was used for protein over-expression and purification (Johnson et al. 1990).

### Knockout construction

The *E. coli* MRE600*ΔyfiA* deletion mutant was obtained from the wild-type MRE600 strain using the standard protocol for one-step inactivation of chromosomal genes (Datsenko and Wanner 2000) with minor modifications.

The kanamycin cassette was amplified from pKD13 (Datsenko and Wanner 2000) plasmid using primers: 5′-ACGGTATGCTGAATTCACCAAGACGGGAAGACAAGAGGTAAAATTTATGATTCCGGGGATCCGTCGACC-3′ and 5′-CGCGTTGGCGATACACTCAATATAAAGGACTACTCTTCTTCAACTTCTTCTGTAGGCTGGAGCTGCTTCG-3′.

The resulting amplicon was used for the gene disruption step as described (Datsenko and Wanner 2000). The polymerase chain reaction (PCR) verification step on KmR transformants was carried out with two flanking locus-specific primers: 5′-TCACACATTTTGACATCAGG-3′ and 5′-AGTGACTTTAGTACAGTACC-3′. The same reaction was performed to check the loss of resistance gene after eliminating the antibiotic resistance gene.

### Growth curves and viable counts

Growth curves of *E. coli* MRE600 and MRE600*ΔyfiA* were carried out in Luria-Bertani (LB) medium and in M9 minimal medium (Sambrook and Russell 2001) at 37°C or during cold shock. To induce cold shock, cultures of *E. coli* MRE600 *wt* and MRE600 *ΔyfiA* grown at 37°C were transferred at 10°C upon reaching OD_600_ = 0.4. Growth was monitored by measuring optical density (OD_600_) and by assaying colony-forming units on LB-plates.

### Two-dimensional gel electrophoresis analysis

Cultures of *E. coli* MRE600 and MRE600*ΔyfiA* grown at 37°C till OD_600_ = 0.6 were transferred to 10°C. Aliquots of both cultures were taken immediately before or after 60 min of cold shock. The cells were then subjected to a freeze–thaw and lysozyme procedure (Ron et al. 1966) and the resulting extracts were fractionated on a sucrose gradient as previously described (Powers and Noller 1990). The fractions containing 70S were pooled and the 70S ribosomes were pelleted by centrifugation in a Sorvall M120 at 80 krpm for 2 h in a S80-AT3 rotor. Ribosomal proteins were obtained from 70S ribosomes by acetic extraction (Hardy et al. 1969) in the presence of 100 mmol/L MgCl_2_. Proteins were then precipitated with two volumes of acetone and dissolved in 100 μL of sample buffer (1 mmol/L *bis*-Tris; 60 mmol/L Urea; 10 mmol/L 1,4-dithio-D-threitol (DTT); 0.05% pararosa aniline). Two-dimensional (2D) gel electrophoresis was performed with a 4–5 pH gradient for the first dimension and a 15% sodium dodecyl sulfate poly acrylamide gel elctrophoresis (SDS-PAGE) as the second dimension, essentially as described (Subramanian 1974; Mets and Bogorad 1974; Madjar et al. 1979). The resulting gels were stained with Comassie blue and then scanned.

### De novo protein synthesis after cold shock

Pulse-labeling experiments were performed on cultures grown in M9 minimal medium (Sambrook and Russell 2001) supplemented with 0.5% glucose and 0.01% yeast extract. MRE600 wt cells and MRE600 *Δyfia* cells were grown to 0.5 A_600_ and then shifted to 10°C. At the indicated times following the temperature shift, 1 mL aliquots from both cultures were pulse-labeled for 30 min with 1 μL of [^35^S] Pro-Mix (7 mCi; 3000 Ci mmol, Amersham) and then chased for 5 min by adding nonradioactive methionine and cysteine to a final concentration of 0.2 mol/L. Two control samples were also pulse-labeled in the same way for 10 min at 37°C before the cold shock. After centrifugation, each cell pellet was resuspended in 200 μL of phosphate buffered saline buffer containing 1 mmol/L DTT and 0.2 mmol/L phenylmethanesulfonylfluoride (PMSF). The cells were then lysed by sonication and the resulting cell extracts were cleared by centrifugation. The total cell extracts thus obtained, normalized for the A_600_ value of the corresponding cell aliquot, were subjected to SDS-PAGE at various concentration (7.5%, 10%, 12.5%, and 15%). The radioactivity incorporated into the proteins was determined with a GS 250 Molecular Imager (Bio-Rad, Hercules, CA).

### Cloning and mutagenesis of *E. coli* gene *yfiA*

The coding region of *E. coli yfiA* gene was amplified by PCR from MRE600 genomic DNA using primers: 5′-CCACGCCATGGCAATGAACATTACCAGCAAAC-3′ and 5′-CGGGATCCCTACTCTTCTTCAACTTCTTC-3′. The PCR product was gel purified, treated with DpnI, and cloned into the pETM11 vector (Dümmler et al. 2005) using NcoI and BamHI restriction sites. *Escherichia coli* BL21 (DE3)/pLYS was transformed by the resulting vector pETM11-PY. The pETM11-PY construct was confirmed by DNA sequencing.

The Val60Cys*_yfiA* mutant was produced using the QuikChange Site-Directed Mutagenesis Kit (Agilent Technologies, Inc., Santa Clara, CA) from Stratagene and the pETM11-pY plasmid as DNA template, with the following mutagenic primers: 5′-CCACTGGCAACCAGACAGCCGTTAGGTGTATTG-3′ and 5′-CCAAGGCCGTACTCCAACTGTAATCAAAAAAGCAA-3′. The resulting construct pETM11-Val60Cys_PY was sequenced to confirm the mutation and transferred in *E. coli* BL21 (DE3)-pLYS by electroporation.

### Purification of PY and PY Val60Cys mutant

The overproduction of protein PY wt or mutant (Val60Cys_PY) was induced by the addition of 1 mmol/L isopropyl-beta-D-1-thiogalactopyranoside to *E. coli* BL21 (DE3)-pLYS cells harboring pETM11-PY or pETM11-Val60Cys_PY, grown in LB medium at 37°C to OD_600_ = 0.4. After transferring the cultures to 20°C for 12 h, the cells were harvested by centrifugation and the pellets were resuspended in Buffer A (25 mmol/L tris-HCl, pH 8.5, 5% glycerol, 100 mmol/L NaCl, 0.025% Nonidet P40) and stored at −80°C. After thawing, the cells were diluted in an equal volume of Buffer B (25 mmol/L tris-HCl, pH 8, 1.3 mol/L NaCl, 5% glycerol, 6 mmol/L β-mercapto-ethanol, 0.1 mmol/L PMSF, 0.1 mmol/L benzamidine), lysed by sonication, and the resulting cell extracts were cleared by centrifugation. The cell extracts were subsequently loaded on a nickel-nitrilotriacetic acid (Ni-NTA) chromatographic column equilibrated in Buffer C (25 mmol/L tris-HCl, pH 8.0, 700 mmol/L NaCl, 5% glycerol, 6 mmol/L β-mercapto-ethanol, 0.1 mmol/L PMSF, 0.1 mmol/L benzamidine). The column, initially washed with the same buffer, was subsequently washed with Buffer D (25 mmol/L tris-HCl, pH 8.0, 300 mmol/L of NaCl, 5% glycerol, 20 mmol/L Imidazole, 6 mmol/L β-mercapto-ethanol, 0.1 mmol/L PMSF, 0.1 mmol/L benzamidine). Protein PY was eluted from the column with Buffer E (25 mmol/L tris-HCl, pH 8.0, 300 mmol/L of NaCl, 5% glycerol, 300 mmol/L imidazole, 6 mmol/L β-mercapto-ethanol, 0.1 mmol/L PMSF, 0.1 mmol/L benzamidine), and the fractions containing protein PY were pooled and dialyzed against Buffer F (25 mmol/L tris-HCl, pH 8.0, 100 mmol/L NaCl, 5% glycerol, 6 mmol/L β-mercapto-ethanol, 0.1 mmol/L PMSF, 0.1 mmol/L benzamidine). To remove the his-Tag sequence from PY protein, 8.4 mg of purified PY was then incubated for 4 h with 0.5 mg of the His-Tag TEV protease (Kapust et al. 2002). At the end of the incubation, the concentration of NaCl was increased to 300 mmol/L and the reaction mixture containing the cleaved PY protein was loaded on a Ni-NTA column equilibrated in Buffer G (25 mmol/L tris-HCl, pH 8.0, 300 mmol/L NaCl, 5% glycerol, 6 mmol/L β-mercapto-ethanol, 0.1 mmol/L PMSF, 0.1 mmol/L benzamidine). The flow-through, containing PY with no His-tag, was concentrated by centrifugation in Amicon ultra-4 centrifugal filter devices at 4 krpm for 40 min at 4°C. Concentrated protein PY was dialysed overnight at 4°C against Buffer H (20 mmol/L tris-HCl, pH 7.1, 100 mmol/L NH4Cl, 1 mmol/L MgCl2, 5% glycerol, 0.1 mmol/L EDTA, 6 mmol/L β-mercapto-ethanol) and stored at −80°C in small aliquots.

### In vitro transcription and mRNA preparation

The templates used for the in vitro synthesis of the various mRNAs were plasmids derived from pTZ18R and pTZ19R (Pharmacia, GE Healthcare Biosciences, Pittsburgh, PA) or pUT7 (Serganov et al. 1997), propagated in and purified from *E. coli* JM109. Namely, these plasmids were pUT*cspA* (Giuliodori et al. 2010), pUT7*cspD* (Spedalieri et al., unpubl. ms.), pUT7*cspB*, puT7*cspE*, pUT7*cspG*, pUT7*cspI* (this study), pTZ19*hupA*, pTZ19*hupB* (Giangrossi et al. 2001), pTZ18*P1infA*, pTZ18*P2infA* (Giangrossi et al. 2007), and pSELECT*hns* (Spurio et al. 1997).

Preparative transcription of the mRNAs with T7 RNA polymerase and mRNA purification were carried out essentially as described (Brandi et al. 1996).

### In vitro translation tests

Before use, each mRNA was denatured at 90°C for 1 min in RNase-free H_2_O and renatured for 15 min at 15°C or 37°C in 20 mmol/L 4-(2-hydroxyethyl)-1-piperazineethanesulfonic acid-KOH (pH 7.5), 10 mmol/L MgCl_2_, 50 mmol/L KCl. In vitro translation tests were carried out essentially as described (Giuliodori et al. 2004, 2007) in 8 mmol/L Mg acetate. The reaction mixture also contained protein PY in the amounts indicated for each experiment.

### 30S and 70S initiation complex formation

Each reaction mixture (30 μL) contained 20 mmol/L tris-HCl, pH 7.7, 80 mmol/L NH_4_Cl, 7 mmol/L Mg acetate, 2 mmol/L DTT, 0.5 mmol/L GTP, 1 μmol/L f[^35^S]Met-tRNA, 1 μmol/L mRNAs, 0.5 μmol/L IF1, 0.5 μmol/L IF2, 1 μmol/L IF3, and 0.5 μmol/L of *E. coli* 30S ribosomal subunit or a 1:1 stoichiometric mixture of 30S and 50S subunits. Protein PY was added to the reaction mixtures at the indicated PY/ribosomal particles ratios. After 30 min of incubation at 15°C, the amounts of ribosome-bound f[^35^S]Met-tRNA was determined by filtering each mixture through nitrocellulose disks.

### Fluorescent labeling of Val60Cys_PY

The PYVal60Cys mutant was labeled with the fluorescent dye Alexa555 essentially as described in Milon et al. (2007). The excess unincorporated fluorophore was separated from the labeled factor (PY_Alexa555) by extensive centrifugation in Amicon ultra-4 centrifugal filter devices (cut off 10 K) at 4 krpm, 4°C.

### Rapid kinetic measurements

All experiments were carried out in 10 mmol/L tris-HCl, pH 7.7, 7 mmol/L Mg acetate, 60 mmol/L NH_4_Cl in a Stopped-flow apparatus Kintek SF-2004 (KinTek Corp., Austin, TX) by mixing at 15°C equal volumes (20 μL) of reactants present in syringe A or syringe B. The final concentration of the molecules used in the various experiments is indicated in the figure legends. Curves were fitted with second-order equation using GraphPad Prism software. In a single experiment, 1000 data points were acquired, usually in logarithmic sampling mode. Each trace is the average of at least 10 different shots. The fluorescence and light scattering values detected at time = 0 were subtracted from each of the 1000 point which constitute each trace.

Binding of PY to naked 30S, 50S, and 70S ribosomes was followed by fluorescent change in PY labeled with Alexa555. Syringe A contained PY_Alexa555, while syringe B contained the 30S, the 50S, or the 70S tight coupled ribosomes. For the release assays, 30S-PY_Alexa555 complexes were rapidly diluted with an equal volume of reaction buffer. For both types of experiments, fluorescence excitation was performed at 552 nm and output monitored using a KV590 nm filter.

Inhibition of 30S initiation complex formation by PY was followed by fluorescence resonance energy transfer (FRET) between fMet-tRNA_fluo (donor) and IF3 labeled with ALEXA 555 (acceptor), prepared as described (Milon et al. 2007). Syringe A contained fMet-tRNA_fluo, IF2, IF1, IF3_ALEXA 555, GTP; syringe B contained 30S ribosomal subunits, GTP and, when required, PY. Excitation was at 465 nm and output monitored using a KV590 nm filter.

For the Light Scattering experiments, 30S subunits with or without increasing amounts of PY (syringe A) and 50S ribosomal subunits (syringe B) were allowed to mix in 10 mmol/L tris-HCl pH 7.7, 5 mmol/L Mg acetate, 60 mmol/L NH_4_Cl; excitation was at 436 nm and output was monitored with no filtering.

## Results

### The *yfiA* gene is dispensable for adaptation at low temperature

A *YfiA* deletion mutant was generated in *E. coli* strain MRE600 as described in Experimental Procedures. The deletion of the gene encoding protein PY from the *E. coli* chromosome was assessed by PCR, as shown in Figure S1A. The mutant strain was also screened for the lack of expression of protein PY by comparing the 2D electrophoretic migration patterns of the proteins extracted from 70S ribosomes purified from wt and mutant cells grown at 37°C or subjected to 60 min of cold shock (Fig. S1B). In agreement with published data (Agafonov et al. 2001), the spot corresponding to protein PY appeared only in the wt MRE600 strain upon cold shock.

**Figure 1:**
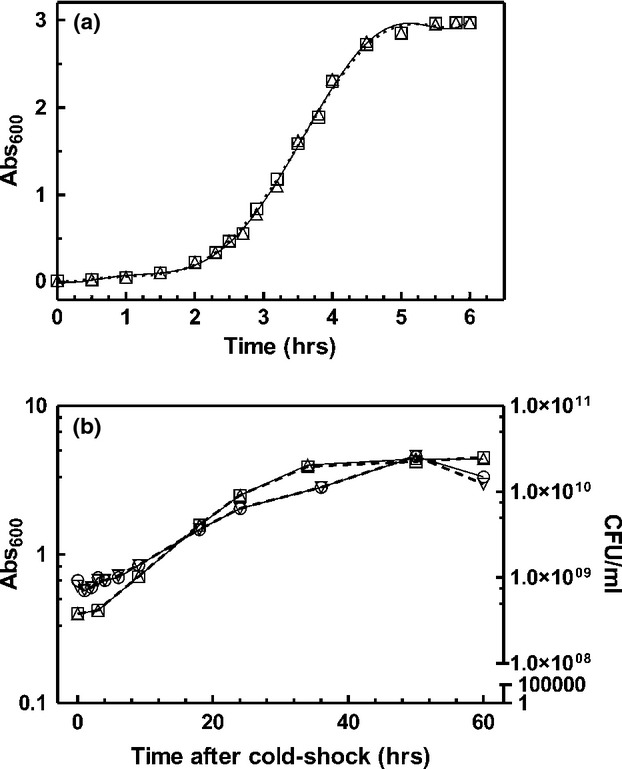
Growth and viability of MRE600 wt and MRE600 *ΔyfiA*. (A) Growth curves of *Escherichia coli* MRE600 wt (□) and *ΔyfiA* cells (Δ) incubated at 37°C in LB medium. (B) Growth curves of wt and *ΔyfiA* strains during cold shock (10°C), monitored by optical density (left axis, MRE600 wt □; MRE600 *ΔyfiA* Δ*)* and number of colony-forming unit (right axis, MRE600 wt ○; MRE600 *ΔyfiA* ∇).

The growth curves of *wt* and *ΔyfiA* strains at 37°C in LB (Fig. 1A) or M9 minimal medium (not shown) show that the two strains have the same doubling time during the exponential phase and level off in the same way when approaching the stationary phase, in agreement with previously reported data (Ueta et al. 2005).

The cold-shock analysis of *wt* and *ΔyfiA* strains was performed in LB medium by rapidly chilling at 15°C cultures grown at 37°C to 0.4 OD_600_. It can be seen (Fig. 1B) that the optical density and the number of colony-forming cells of both cell cultures started to increase after about 4 h of cold shock and reached the maximum after 40 h. More importantly, no difference was observed between wild-type and *ΔyfiA* mutant growth curves upon cold shock. A similar result was obtained when studying *ΔyfiA* mutants prepared from other *E. coli* strains (W3110, C-1a, and BW25113; not shown).

### PY affects gene expression but is not responsible for the reduction of bulk protein synthesis at the onset of the cold stress

**Figure 2:**
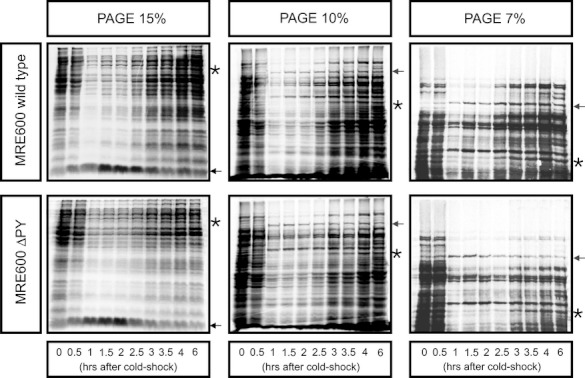
De novo translation following cold shock in the wt and *ΔyfiA* strains. Protein synthesis of *Escherichia coli* MRE600 wt and MRE600 *ΔyfiA* was compared by pulse-chase experiments performed immediately before cold shock (time 0) or at different time intervals after a temperature shift from 37°C to 10°C. To highlight possible differences, the same samples were run in gels containing three different concentrations of polyacrylamide, as indicated. The black asterisk specifies the migration of the same protein band in the various gels. The black and the gray arrows indicate the bands corresponding to CspA and an unidentified cold-shock protein, respectively. Further details are given in Experimental Procedures.

To compare protein expression in *E. coli* MRE600 wt and MRE600 *ΔyfiA,* these strains were grown at 37°C in M9 minimal medium and shifted to 10°C when OD_600_ = 0.8. At the indicated times (Fig. 2), an aliquot of each culture was labeled with [^35^S] pro-mix and then chased with nonradioactive Met and Cys. Samples of each aliquot, corresponding to equal OD units, were subjected to SDS-PAGE analysis at 7%, 10%, and 15% acrylamide concentrations and the radioactivity incorporated into proteins was quantified by exposure in a molecular imager.

As seen in Figure 2, the intensity of the bands, which is proportional to the amount of protein synthesized in each labeling time window, is drastically reduced in both wt and mutant strains for the majority of the proteins immediately after the temperature downshift and starts to increase again only ≅3 h later. This result indicates that PY is not responsible for the block of protein synthesis during cold shock, at variance with what had been suggested (Vila-Sanjurjo et al. 2004). However, a quantitative analysis of the gels (Fig. S2) seems to point out a difference in the behavior of the two strains. In fact, the reduction of bulk protein synthesis is moderately stronger in the wt than in the deletion mutant, and the resumption of translation at the exit of the adaptation phase seems to be more rapid and efficient in the wt strain than in *ΔyfiA*. Interestingly, also the induction of some cold-shock protein is different in the two strains. For instance, in the deletion strain the band indicated by the gray arrow (Figs. 2 and S2) appears immediately after cold shock and disappears rapidly, whereas its expression in the wt strain is delayed and lasts longer. On the other hand, induction of CspA (black arrow) and other unidentified cold-shock proteins is similar in the two types of cells (Figs. 2 and S2).

Overall, these data suggest that PY could partially affect bulk translation and influence the timing of the cold-shock induction of some proteins. If correct, this hypothesis would reconcile the inhibition of translation caused by PY observed in vitro by several groups (Agafonov et al. 2001; Vila-Sanjurjo et al. 2004; Sharma et al. 2010) with the increased translational activity described for some mRNAs during cold shock (Gualerzi et al. 2003; Giuliodori et al. 2004).

### PY inhibition depends on temperature and type of mRNA

The results of the experiments described in the above section suggest that protein PY may be able to influence to different extents the synthesis of different proteins. To verify this hypothesis, we performed in vitro translation tests using various mRNAs of different classes, namely cold-shock (cs), cold-tolerant (ct), and non–cold-shock (non-cs) mRNAs. Among the first class, we selected *cspA* mRNA and *hns*, encoding the most abundant cold-shock protein of *E. coli* CspA (Goldstein et al. 1990) and the nucleoid-associated protein H-NS (La Teana et al. 1991), respectively. Furthermore, we tested two other cs transcripts, *cspI* and *cspG mRNAs,* belonging to the *E. coli csp* family (Yamanaka et al. 1998). *P1infA* and *P2infA* mRNAs are two alternative transcripts encoding initiation factor IF1: *P1infA* mRNA is preferentially produced and translated during cold shock, while *P2infA* mRNA can be considered a non-cs transcript, being mainly transcribed at 37°C (Giangrossi et al. 2007). *HupA* and *cspD* mRNAs represent two other typical non-cs transcripts, the first encoding the α subunit of the nucleoid-associated protein HU (Giangrossi et al. 2002) and the second encoding CspD, a protein that, unlike its paralog CspA, is expressed only at 37°C (Yamanaka and Inouye 1997). As ct mRNAs, we selected *HupB* and *cspE* mRNAs encoding the β subunit of HU (Giangrossi et al. 2002) and protein CspE (Xia et al. 2001), respectively, as they are synthesized at both low and high temperature. Finally, the *027-C* mRNA is the transcript of a semi-synthetic gene designed for the optimization of in vitro translation experiments to be performed at 37°C with various organisms (Brandi et al. 2007).

Translation of such mRNAs was studied in the presence of increasing amounts of purified PY, using crude cellular extracts prepared from cells not subjected to cold shock (control S30) or subjected to 120 min of cold shock (csS30). These experiments were carried out: (i) at 37°C with control S30 to reproduce the milieu existing when cells grow under physiological conditions; (ii) at 15°C with control S30 to reproduce the conditions existing at the onset of the cold shock when translation occurs in the absence of the cs *trans*-acting factors, which are expressed only at a later stage of cold adaptation (Brandi et al. 1999; Giuliodori et al. 2004); (iii) at 15°C with csS30, when all the cs *trans*-acting factors are present. The points of each experiment were normalized by taking the amount of protein synthesized in the absence of PY as 100%.

**Figure 3:**
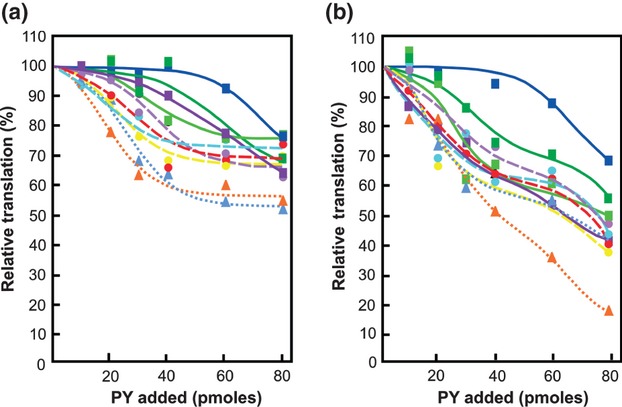
Effect of PY on the translation of various mRNAs at 15°C. Translation of cold-shock, cold-tolerant, and non–cold shock mRNAs was performed in 30 μL reaction with 7 μL of control S30 (A) or cold shock S30 (B) programed with 30 pmoles each of the indicated mRNAs (*cspA* mRNA 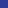, *hupB* mRNA 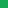, *P1infA* mRNA 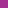, *cspE* mRNA 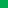, *P2infA* mRNA 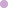, *cspD* mRNA 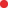, *cspI* mRNA 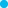, *hupA* mRNA 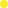, *cspG* mRNA 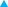, *027-C* mRNA 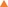) under the conditions described in Experimental Procedures. Furthermore, each reaction mixture contained the amounts of PY indicated in the abscissa. Incubation was performed for 120 min at 15°C. The results are presented as percent of the level of product obtained in the absence of PY set at 100%.

The results show that at 15°C, PY inhibited to different extents the translation of all mRNAs examined, with both control S30 (Fig. 3A) and csS30 (Fig. 3B), whereas it did not affect translation at 37°C (not shown), in agreement with previous results (Vila-Sanjurjo et al. 2004). The inhibition observed with the control S30 seems to affect primarily translation of the semi-synthetic *027-C* mRNA and *cspG* mRNA (45% inhibition), whereas translation of the other mRNAs was either marginally affected (20–30% inhibition) or affected only at the highest concentration of PY (*cspA*, *hupB*, and *P1infA* mRNAs). The inhibition observed in the presence of the csS30 extract seems overall more pronounced (Fig. 3B) than that with the control S30 extract. In fact, under these conditions, translation of most mRNAs was inhibited by PY between 40% and 50%, with the exception of *027-C* mRNA (80% inhibition) and *cspA* mRNA, which was repressed only at the highest PY concentration. The fact that inhibition was found to be somewhat stronger with the cold-shock cellular extract could be explained either by the presence of endogenous PY associated with the cs ribosomes or by the presence of other unidentified cs factors capable of reinforcing the inhibitory activity of this protein.

Taken together, these results demonstrate that PY reduces translation in a temperature- and mRNA-dependent manner. This activity is rather modest under the conditions tested, in agreement with the in vivo data which do not support a central regulative role of protein PY during cold stress.

### PY inhibits mainly the initiation phase of translation

Previously published data suggest that PY may inhibit two steps of the translation process, namely initiation (Vila-Sanjurjo et al. 2004) and elongation (Agafonov et al. 2001). To further clarify which translational step is affected by PY, translation of the various mRNAs was tested at low temperature in the presence of increasing amounts of PY using 70S ribosomes and S100 postribosomal supernatants prepared from control S30. Subsequently, the same 70S ribosomes were dissociated into 30S and 50S ribosomal subunits to study the formation of 70S initiation complexes (70S IC) at 15°C. In these experiments, the 30S subunits were incubated with IF1, IF2, IF3, mRNA, f[^35^S]Met-tRNA, and 50S subunits in the presence of increasing concentrations of PY and the amount of f[^35^S]Met-tRNA bound in the 70S IC was determined by nitrocellulose filtration.

**Figure 4:**
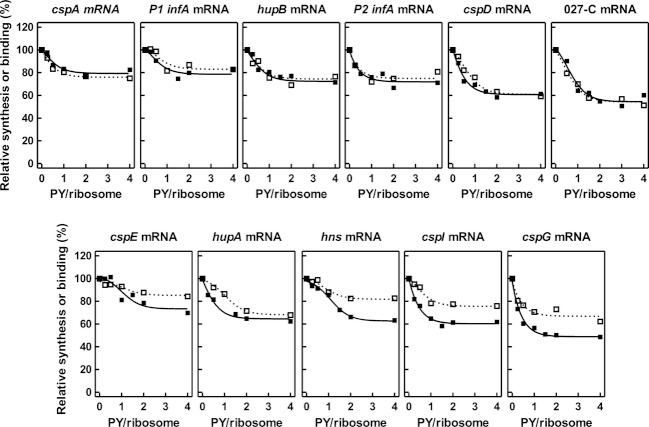
Comparison between in vitro protein synthesis and 70S initiation complexes formation. All reactions were carried out at 15°C as a function of the increasing PY/ribosome ratio indicated in the abscissa. The translation tests (▪) were performed in 30 μL reaction with 30 pmoles of high salt washed 70S ribosomes, 15 pmoles of IF1, IF2, IF3, 2 μL of S100 postribosomal supernatant, and 30 pmoles of the indicated mRNAs. The 70S IC (□), programed with the indicated mRNAs, were prepared under the conditions described in Experimental Procedures. The results of the translation tests and the filter binding assays are presented as percent of the amount of protein synthesized in the absence of PY and of the level of f[^35^S]Met-tRNA bound in the absence of PY, set at 100%. The amount of radioactivity remaining on the filters in the absence of mRNA was taken as representing the background and subtracted from each point.

The effect of PY on both the entire translational process and the single initiation step is shown in Figure 4. The results obtained confirm the mRNA-dependent inhibition by PY (compare *cspA* with 027-C or *cspD* mRNAs) and, in addition, indicate that this inhibition takes place mainly at the level of initiation complex formation. In fact, in most of the cases the curves describing the effect of PY on translation (closed symbol) and 70S IC formation (open symbol) are perfectly (*cspA*, *027-C*, *cspD*, *P1infA*, *P2infA*, *hupB* mRNAs) or almost perfectly (*hupA* and *cspE* mRNAs) superimposable. On the other hand, the behavior of *cspE*, *cspG*, *hns*, and *cspI* mRNAs is different from that of the other transcripts; in these cases, it can be hypothesized that further inhibition by PY occurs at the level of elongation. It remains to be elucidated why and how PY could affect the translation elongation step of only some and not other mRNAs.

### PY binds preferentially to the 30S subunits and increases the association rate of the ribosomal subunits

**Figure 5:**
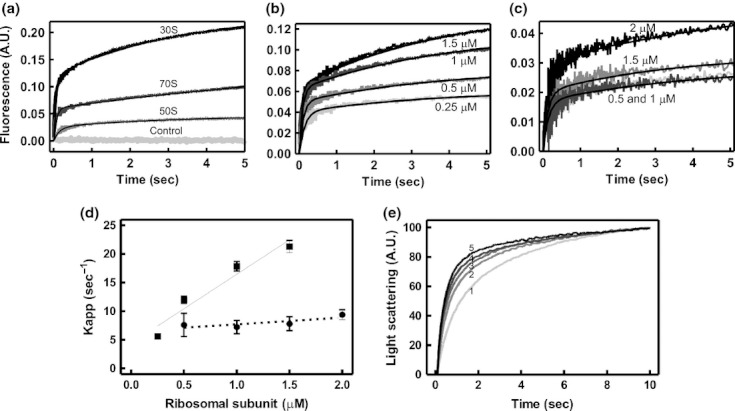
Binding of PY to the 30S, 50S, and 70S ribosome and stimulation of subunit association. (A) Time course of PY_Alexa555 binding to 30S subunits, 50S subunits, or 70S tight coupled ribosomes. The reaction was performed at 15°C in the stopped-flow apparatus by mixing 0.1 μmol/L PY_Alexa555 with 0.5 μmol/L 30S, 50S, or 70S ribosomes. Control measurement was performed by mixing the fluorescently labeled protein with the reaction buffer. (B) Time course of 0.1 μmol/L PY_Alexa555 binding to 0.25, 0.5, 1, and 1.5 μmol/L of 30S subunits. (C) Time course of 0.1 μmol/L PY_Alexa555 binding to 0.5, 1, 1.5, and 2 μmol/L of 50S subunits. (D) Concentration dependence of *k*_app1_ from the binding of PY to the 30S subunits (▪) or the 50S subunits (•). The *y*-axis intercept gives the value of the backward rate constant *k*_off,_ while the slope of the lines corresponds to the forward rate constant *k*_on_ of the reactions. Standard deviations were calculated from at least 10 different time courses. (E) Association of 30S and 50S ribosomal subunits studied by light scattering. 30S subunits (0.1 μmol/L) and 50S subunits (0.1 μmol/L) were rapidly mixed in the stopped-flow instruments at 15°C in the absence (tracing 1) or in the presence of 0.1 μmol/L (tracing 2), 0.2 μmol/L (tracing 3), 0.5 μmol/L (tracing 4), and 1 μmol/L (tracing 5) of protein PY. Further details are given in Experimental Procedures.

To understand the mechanism of action of PY, binding of this protein to the ribosomal subunits and to the 70S ribosome was studied at 15°C by monitoring the fluorescence change of an engineered PY in which the native Valine in position 60 was replaced by a Cysteine (Val60Cys_PY) to allow the labeling of the protein with fluorophore Alexa555 (PY_Alexa555). Control experiments showed that Val60Cys_PY has an activity comparable to the native PY (data not shown) and that an increase in fluorescence can be observed only when the PY_Alexa555 is mixed with the ribosome (Fig. 5A).

As the experimental curves obtained by mixing PY with the 30S subunit, the 50S or the 70S ribosome (Fig. 5A) can be fitted with the function Y = A0 + A1 × exp (−*k*_app1_ × x) + A2 × exp (−*k*_app2_ × x), a two-step binding of PY to all macromolecules tested may be hypothesized. In the above mentioned equation, *k*_app1_ and *k*_app2_ refer to the apparent rate constants of the first and second phase and A1 and A2 to the respective amplitudes.

To extrapolate the association (*k*_on_) and dissociation (*k*_off_) rate constants of the binding reactions, the values of *k*_app1_ and *k*_app2_ were determined as a function of increasing 30S and 50S subunit concentrations (Fig. 5B and C, respectively) and plotted (Fig. 5D). This approach yielded *k*_on1_ = 12.1 ± 2.1 μmol/L per sec and *k*_off1_ = 4.0 ± 2.1 sec^−1^ for the binding to the 30S subunits (Fig. 5D, squares), and *k*_on1_ = 1.2 ± 0.6 μmol/L per sec and *k*_off1_ = 6.5 ± 0.8 sec^−1^ for the binding to the 50S subunits (Fig. 5D, circles), whereas the values of *k*_app2_ (≅0.3 s^−1^ for both 30S and 50S) were shown to be concentration independent (Fig. S3A). This outcome suggests that the second phase that follows the first binding step likely corresponds to a slow rearrangement of PY on the ribosomal subunits.

To calculate the *k*_off2_, we examined the fluorescence changes resulting from the rapid dilution of the 30S-PY_ALEXA555 complex and the 50S-PY_ALEXA555 complex (Fig. S3B). As *k*_app1_ >> *k*_app2_, we can assume *k*_app2_ = *k*_on2_ + *k*_off2_ and derive *k*_on2_ ≅0.23 knowing that the calculated *k*_off2_ is ≅0.07 sec^−1^ for both reactions. From the rate constants, the dissociation constants of the two phases (*k*d_1_ and *k*d_2_) can be calculated, which in turn can be used to derive the dissociation constant of the binding reactions (*k*d = *k*d_1_ × *k*d_2_), corresponding to 83 nmol/L for 30S-PY and 1.26 μmol/L for 50S-PY.

In conclusion, these experiments show that PY binds rapidly and tightly to the 30S ribosomal subunits and very slowly and weakly to the 50S subunits. As for the binding to the 70S ribosome, it probably occurs with an intermediate affinity.

Finally, to determine whether the PY binding to the 30S subunits is reflected in a change in the association rate of the small and large subunits, formation of idle 70S ribosomes was studied following the increase in light scattering at 15°C. The result of the experiment clearly shows that PY accelerates the kinetics of 70S ribosome formation about two times (Fig. 5E).

### PY influences the formation of the 30S initiation complex

**Figure 6:**
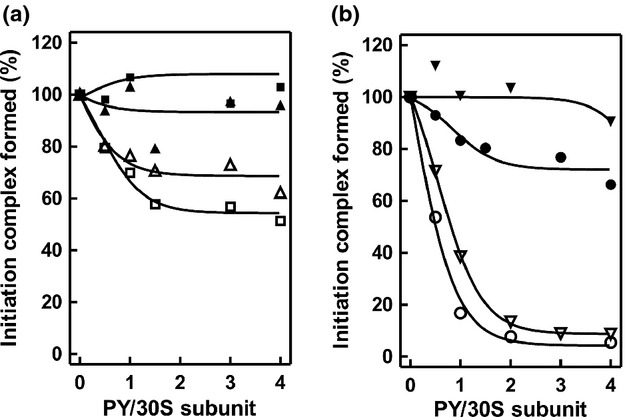
30S and 70S initiation complexes formation in the presence of PY. The initiation complexes were formed at 15°C as described in Experimental Procedures with *cspG* mRNA and *027-C* mRNA (A) or with *cspA* mRNA (B) as a function of the increasing PY/30S ratios indicated in the abscissa. The results of the assays are presented as percentages, setting the level of initiation complex formed in the absence of PY to 100%. (A) PY was added to the reaction mixture either simultaneously with IF1, IF2, IF3, mRNA, f[^35^S]Met-tRNA, 30S and 50S subunits (*cspG* mRNA Δ, *027-C* mRNA □) or after the same components were incubated for 15 min at 15°C to preform 70S initiation complexes (*cspG* mRNA ▲, *027-C* mRNA ▪). (B) The indicated amount of PY was either prebound to the 30S (▽) and the 30S + 50S subunits (○) for 15 min before the addition of IF1, IF2, IF3, *cspA* mRNA, f[^35^S]Met-tRNA or added simultaneously with the other components to the reaction mixture containing the 30S subunits (▼) or the 30S + 50S subunits (•). The amount of radioactivity remaining on the filters in the absence of mRNA was taken as representing the background and subtracted from each point.

To further clarify the reason for the PY-dependent inhibition of translation initiation, formation of the initiation complexes was tested by changing the order in which protein PY was added to the reaction mixture. The first set of experiments was performed at 15°C under the conditions in which PY inhibition in the translational tests was stronger, that is with *cspG* mRNA and *027-C* mRNA. Increasing amounts of protein PY were added to the reaction mixtures either simultaneously with IF1, IF2, IF3, mRNA, f[^35^S]Met-tRNA, 30S and 50S subunits or after the same components were preincubated for 15 min at 15°C to preform 70S initiation complexes. Our results (Fig. 6A) demonstrate that the protein inhibits the reaction only if added before the 70S initiation complexes are formed. Therefore, PY cannot destabilize the f[^35^S]Met-tRNA once this is correctly positioned in the 70S ribosome.

In another series of experiments (Fig. 6B), PY activity was investigated using *cspA* mRNA as template. With this transcript, the 70S complex was only slightly inhibited (20%) when formed in the presence of PY (closed circles), whereas it was strongly inhibited (>80%) when PY was mixed with the 30S and 50S subunits before the addition of IFs and mRNA (open circles). The same experiment was repeated also in the absence of the 50S subunits (Fig. 6B). Also in this case, PY did not influence the reaction when added to the mixture with the other components (closed triangles), whereas it strongly inhibited the 30S IC formation when prebound to the 30S subunits (open triangles).

**Figure 7:**
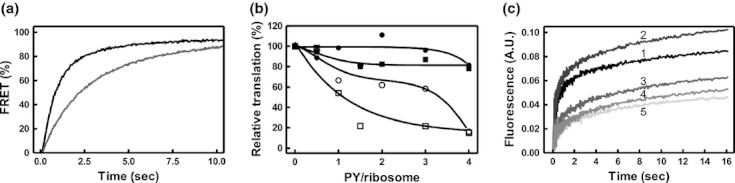
PY influences the formation of the 30S initiation complex. (A) Binding of IFs and fMet-tRNA to the 30S subunit was studied at 15°C by monitoring the FRET signal between fMet-tRNA_fluo and IF3_Alexa555 in the absence (black) or in the presence (gray) of PY. Syringe A contained 0.4 μmol/L fMet-tRNA_fluo, 0.4 μmol/L IF2, 0.4 μmol/L IF1, 0.4 μmol/L IF3_ALEXA 555, 0.5 mmol/L GTP; syringe B contained 0.2 μmol/L of 30S ribosomal subunits, 0.5 mmol/L GTP, and 2 μmol/L PY. (B) In vitro translation of *cspA* mRNA at 15°C (squares) and at 37°C (circles) was carried out as a function of the increasing PY/30S ratios indicated in the abscissa. At both temperatures, protein PY was either added to preformed 30S IC (▪,•) prepared as described in Experimental Procedures or incubated with the 30S for 15 min before the addition of *cspA* mRNA, IF1, IF2, IF3, and f[^35^S]Met-tRNA (□,○). Elongation of the 30S IC thus formed was carried out in 30 μL reaction by adding the 50S subunits, 2 μL of the S100 postribosomal supernatant, 0.5 mmol/L ATP, 0.2 mmol/L GTP, 0.025 mg/mL pyruvate kinase, 0.2 mmol/L PEP, and 0.2 mmol/L amino acid mix. (C) The interaction of PY_Alexa555 (0.1 μmol/L) with the 30S subunit, with various combinations of prebound ribosomal ligands, was monitored at 15°C in the stopped-flow instrument. Tracing 1: 0.1 μmol/L 30S subunit alone; tracing 2: 30S subunit (0.1 μmol/L) preincubated with IF1 (1 μmol/L); tracing 3: 30S subunit (0.1 μmol/L) preincubated with IFs (0.2 μmol/L) and GTP (0.5 mmol/L); tracing 4: 30S subunit (0.1 μmol/L) preincubated with IFs (0.2 μmol/L), GTP (0.5 mmol/L), and *cspE* mRNA (0.2 μmol/L); tracing 5: 30S IC prepared by mixing 30S subunit (0.1 μmol/L), IFs (0.2 μmol/L), GTP (0.5 mmol/L), *cspE* mRNA (0.2 μmol/L), and fMet-tRNA (0.2 μmol/L). Further details are given in the text and in Experimental Procedures.

These results suggest that protein PY is able to bind to the 30S ribosomal subunit but not to the 30S initiation complex and that, once bound, it affects the binding of the other ribosomal ligands. To test this hypothesis, the effect of PY on the kinetics of fMet-tRNA and IFs binding to the 30S subunits was studied by fluorescence stopped-flow analysis (Fig. 7A) making use of the FRET signal between fMet-tRNA_fluo as donor and IF3_ALEXA555 as acceptor (Milon et al. 2007). In this experiment, syringe A contained the 30S ribosomal subunit in the presence or in the absence of PY, while syringe B contained fMet-tRNA_fluo, IF1, IF2-GTP, and IF3_Alexa555. Our data indicate that the presence of PY slows down the binding of fMet-tRNA and IFs to the 30S subunit about two times.

To confirm the result of these experiments, we performed in vitro translational tests (Fig. 7B) at 15°C and 37°C using *cspA* mRNA, 30S and 50S ribosomal subunits, IFs, and S100 postribosomal supernatant. Again, a strong inhibition (≅80% and ≅40% at a PY/30S subunit ratio of 2:1) was observed when protein PY was prebound to the 30S subunits, before the other components were added. On the other hand, if PY was added to the reaction after preincubating for 15 min *cspA* mRNA, 30S subunit, IFs, and S100 postribosomal supernatant, inhibition was reduced (20% inhibition at 15°C and <10% inhibition at 37°C). Note that the stronger inhibition at 15°C than at 37°C confirms that this protein is able to affect translation mainly at low temperature.

The scarce inhibition observed when PY is not prebound to the 30S subunits probably implies that this protein does not compete efficiently with the other ligands (IFs, mRNA, and fMet-tRNA) for the binding to the 30S. To verify our supposition, the fluorescence change in PY_Alexa555 was monitored at 15°C in the presence of various combinations of ribosomal ligands prebound to the 30S subunit (Fig. 7C). All time courses recorded have two exponential phases and the rate of PY association did not change with any condition tested. However, the amplitude of the curves differs manifestly depending on the ligand(s) bound to the 30S. In fact, while the maximum amplitude of the fluorescence change was ≅0.08 with the 30S subunit alone, it slightly increased to ≅0.10 with IF1-30S and almost halved with the 30S-IFs, the 30S-mRNA-IFs complex, and the 30S IC. In the presence of these ligands the fluorescence increase, which is reduced probably because of the incapacity of PY to bind complete or almost complete 30S IC, likely corresponds to the binding of PY to free 30S subunits, which are expected to be quite abundant at 15°C.

## Discussion

Previous studies have shown that one of the main mechanisms by which cold-stressed cells attain an increased expression of the cold-shock genes is by a modification of the translational apparatus that leads to the selective stimulation of cold-shock mRNA translation and to the repression of “non–cold-shock” gene expression at low temperature (Brandi et al. 1996; Goldenberg et al. 1997; Giuliodori et al. 2004). It has been postulated that PY could be responsible for the translational repression at the onset of cold adaptation (Agafonov et al. 2001; Vila-Sanjurjo et al. 2004). Thus, understanding the role and function of PY represents a key step in understanding the mechanism responsible for the reprogramming of gene expression after cold shock. In this study, by comparing the cold-shock response in a wt *E. coli* strain (MRE600) and its isogenic strain lacking the gene encoding for protein PY (*yfiA*) (MRE600 Δ*yfiA*), we demonstrate that neither the recovery from the cold stress (Fig. 1) nor the reduction in protein synthesis immediately after the shift to 10°C (Fig. 2) depends on PY. Therefore, in contrast to the previous claims (Agafonov et al. 2001; Vila-Sanjurjo et al. 2004), our data demonstrate that protein PY does not play a relevant role after cold stress and is not responsible for the shutdown of bulk translation at the onset of the cold shock; this event must be due to other molecular mechanisms, for example a reduction in the capacity of the ribosome to initiate protein synthesis. In fact, initiation is the stage of translation in which most of the regulatory events takes place. The correct codon–anticodon interaction and recognition of the mRNA translation initiation region (TIR) are crucial steps of initiation that may be strongly affected by the secondary structures of the mRNAs in terms of kinetics and efficiency (de Smit and van Duin 2003; Marzi et al. 2007). As low temperature can stabilize alternative RNA secondary structures (Giuliodori et al. 2010), we may speculate that the drastic reduction in bulk translation could be due, at least in part, to unfavorable mRNA structures that impair translation. This is probably one of the reasons for the stringent requirement of RNA chaperones and helicases during cold shock (Gualerzi et al. 2003). Moreover, the increased level of initiation factor IF3 during cold shock may further reduce the synthesis of the non–cold-shock proteins, discriminating against the formation of productive 70S initiation complexes programmed with these types of transcripts (Giuliodori et al. 2007).

The fact that PY does not act as a general inhibitor of the protein synthesis in vivo raises the questions of whether this protein is able to reduce the expression of at least some specific protein and what is the mechanism which allows the cold-shock mRNAs to bypass this inhibition. Quantitative analysis of the bands which correspond to the proteins de novo synthesized after cold shock in the cells (Fig. S2) seems to indicate that PY could indeed partially diminish bulk translation and influence the timing of cold-shock induction of some proteins. The tests carried out in this study further demonstrate that at low temperature PY is able to reduce translation of some mRNAs (Fig. 3A and B). However, the extent of inhibition is rather modest, in agreement with the in vivo data, and do not support a central role of protein PY during cold stress. Although translation of cold-shock mRNAs is also affected, translation of non–cold-shock mRNAs seems to be more susceptible to the presence of PY, in particular under the conditions mimicking the cellular milieu existing immediately after the temperature downshift. Finally, as translation of *cspA* mRNA is basically insensitive to the presence of PY, both in vivo and in vitro, we can conclude that this protein does not hinder the accumulation of the main cold-shock protein of *E. coli*.

Vila-Sanjurjo et al. (2004) have shown that PY occupies the P site of the ribosome and inhibits the mRNA-dependent binding of fMet-tRNA when preincubated with the 70S ribosome. As PY stabilizes 70S ribosomes against dissociation (Agafonov et al. 2001; Vila-Sanjurjo et al. 2004), and bacteria commonly require free 30S and 50S subunits to initiate protein synthesis (Brandi et al. 2008), it remained to be elucidated whether the inhibition of fMet-tRNA binding was caused indirectly by PY-induced stabilization of the 70S ribosomes or directly by PY blockage of 30S initiation complex formation.

The present results show that PY affects directly the initiation of translation at low temperature (Fig. 4). In fact, the stopped-flow experiments demonstrate that PY binds strongly to and dissociate very slowly from the 30S ribosomal subunit (Fig. 5A–D) in agreement with the finding of Agafonov et al. (1999) who studied the association of PY with the 30S subunit and the 70S ribosome by an ultrafiltration binding test. Once bound, PY sequesters the 30S and 50S subunits by stimulating their association in idle 70S monomers (Fig. 5E), thereby diminishing the number of ribosomal subunits able to participate in the formation of 70S initiation complexes (Fig. 6B). However, PY cannot destabilize the fMet-tRNA once this is correctly positioned in the 70S IC (Fig. 6A).

Agafonov et al. (2001) concluded from their analysis that the main stage of translation inhibited by PY is the aminoacyl-tRNA binding during elongation. Our results confirm that PY has an effect also on the translation elongation process, at least with some mRNAs (Fig. 4), but do not support the preferential inhibition of this step.

The fact that the extent of PY inhibition increases dramatically when this protein is preincubated with the 30S subunits or the 30S + 50S subunits (Figs. 6B and 7B) seems to indicate that PY competes with other ribosomal ligands for binding to the 30S subunits, as suggested also from the position of PY in the crystal structure of the 70S ribosome (Vila-Sanjurjo et al. 2004; Polikanov et al. 2012). The present results imply that the PY position on the 30S subunit possibly clashes with that of the initiator tRNA and/or one or more initiation factors (Fig. 7A and C), in agreement with Vila-Sanjurjo et al. (2004) and Polikanov et al. (2012). On the other hand, our stopped-flow data (Fig. 7C) do not seem to support the hypothesis proposed by Vila-Sanjurjo et al., that PY may hinder IF1 binding to the 30S subunit. However, given that our consideration is based on the lack of a fluorescence decrease upon PY binding to the 30S-IF1 complex, it is not inconceivable that a more direct analysis of the possible IF1-PY competition could lead to a different conclusion. Finally, as inhibition is rather modest when tRNA, mRNA, and IFs are free to compete with PY during the formation of the initiation complexes (Figs. 6 and 7B), we can postulate that the reaction that leads to the formation of the initiation complexes is probably faster and/or more efficient than the binding of PY to the small subunit, at least with some mRNAs.

Taken together our findings suggest a new model which can explain the role of PY during cold shock. Upon lowering the temperature, protein synthesis diminishes possibly because of unfavorable mRNA secondary structures capable of reducing the rate of the initiation complex formation. PY would bind to a fraction of the free 30S subunits derived from this cold-induced polysome dissociation (Uchida et al.^1970^; Jones and Inouye 1996), thereby stimulating their joining with 50S subunits to form inactive 70S monomers. As resumption of protein synthesis at the exit of the adaptation phase seems more efficient in the presence of PY (Figs. 2 and S2), we support the hypothesis that PY could protect the unused subunits from degradation during the environmental stress (Maki et al. 2000; Vila-Sanjurjo et al. 2004). However, no difference was observed between wild-type and *ΔyfiA* mutant growth curves upon cold shock in all strains tested (MRE600, W3110, C-1a, and BW25113), regardless the partial or full activity of the ribosome degradation pathway. PY would cause also the partial inhibition of the translation of some mRNAs, while those amenable to initiate efficiently translation in the cold by rapidly forming 30S initiation complexes, such as *cspA* mRNA, would not be affected by the PY activity.

## Supporting Information

**Figure S1.** Assessment of MRE600 *ΔyfiA* mutation. (A) PCR of *yfiA* locus. Lane 1: 100 bp DNA ladder (Fermentas, Thermo Fisher Scientific, Inc., Waltham, MA); lane 2: amplification of the *Escherichia coli* wt *yfiA* locus; lane 3: amplification of the *yfiA* locus after recombination with a kanamycin cassette (*yfiA**::**kan*); lane 4: amplification of the *yfiA* locus after kanamycin cassette excision (*ΔyfiA*). (B) Two-dimensional gel electrophoresis analysis of 70S ribosomal proteins isolated from *E. coli* MRE600 wt (left) and *ΔyfiA* cells (right) grown at 37°C (upper panels) or subjected to 60 min cold shock (lower panels). The spot corresponding to protein PY is circled. Further details are given in Experimental Procedures.

**Figure S2.** In vivo protein expression after cold shock. Synthesis of the proteins in MRE600 wt (gray lines) or MRE600 *ΔyfiA* (black lines) was followed by giving pulses of [^35^S]-Promix, as described in Experimental Procedures, immediately before (time 0) or at the times following cold shock indicated in the graphs. After chasing with an excess of unlabeled Met and Cys, the samples were processed for the electrophoretic separation at 7%, 10%, and 15% acrylamide concentrations and for the determination of the radioactivity using a Molecular Imager (Bio-Rad GS 250). The peaks in the graphs correspond to the intensity, expressed as “arbitrary units,” of the same point of each band present in the lanes of the various gels. The black and gray arrows indicate the pick corresponding to CspA and another unidentified cold-shock protein, respectively.

**Figure S3.** (A) Concentration dependence of *k*_app2_ from the binding of PY to the 30S subunit (▪) or the 50S subunit (•). The *y*-axis intercept gives the value of the backward rate constant *k*_off_, while the slope of the lines corresponds to the forward rate constant *k*_on_ of the reactions. Standard deviations were calculated from at least 10 different time courses. (B) Time courses of PY dissociation from 30S subunit (gray) or 50S subunit (black) monitored by PY_Alexa555 fluorescent change; 0.6 μmol/L of PY_Alexa555 and 0.2 μmol/L of 30S or 50S subunits were preincubated at 15°C and then rapidly mixed with the reaction buffer. Further details are given in the text and in Experimental Procedures.
