# Supplementary material for: Role of the ribosome-associated protein PY in the cold-shock response of Escherichia coli
Source: Microbiologyopen. 2013 Feb 19;2(2):293–307. doi: 10.1002/mbo3.68 (PMC3633353; doi:10.1002/mbo3.68)
Supplement: Supplementary file 1 [file mbo30002-0293-SD1.pdf]

# Supporting Information

# Role of the ribosome-associated protein PY in the cold-shock response of *Escherichia coli*

*Fabio Di Pietro[1], Anna Brandi[1], Nadire Dzeladini[1], Attilio Fabbretti[1], Thomas Carzaniga[2], Lolita Piersimoni[3] ,Cynthia L. Pon[1] and Anna Maria Giuliodori[1]*

1 Laboratory of Genetics, School of Biosciences and Biotechnology, University of Camerino, 62032 Camerino (MC), Italy
2 Dipartimento di Bioscienze, Università degli Studi di Milano, 20133 Milan, Italy.
3Present address: Department of Molecular, Cellular and Developmental Biology, University of Michigan, Ann Arbor, Michigan 48109, USA

Corresponding author:

Anna Maria Giuliodori
Laboratory of Molecular Biology and Biotechnology
School of Biosciences and Biotechnology
University of Camerino,
62032 Camerino (MC), Italy
Tel +39 0737 403251
Fax +39 0737 403290
e-mail: annamaria.giuliodori@unicam.it

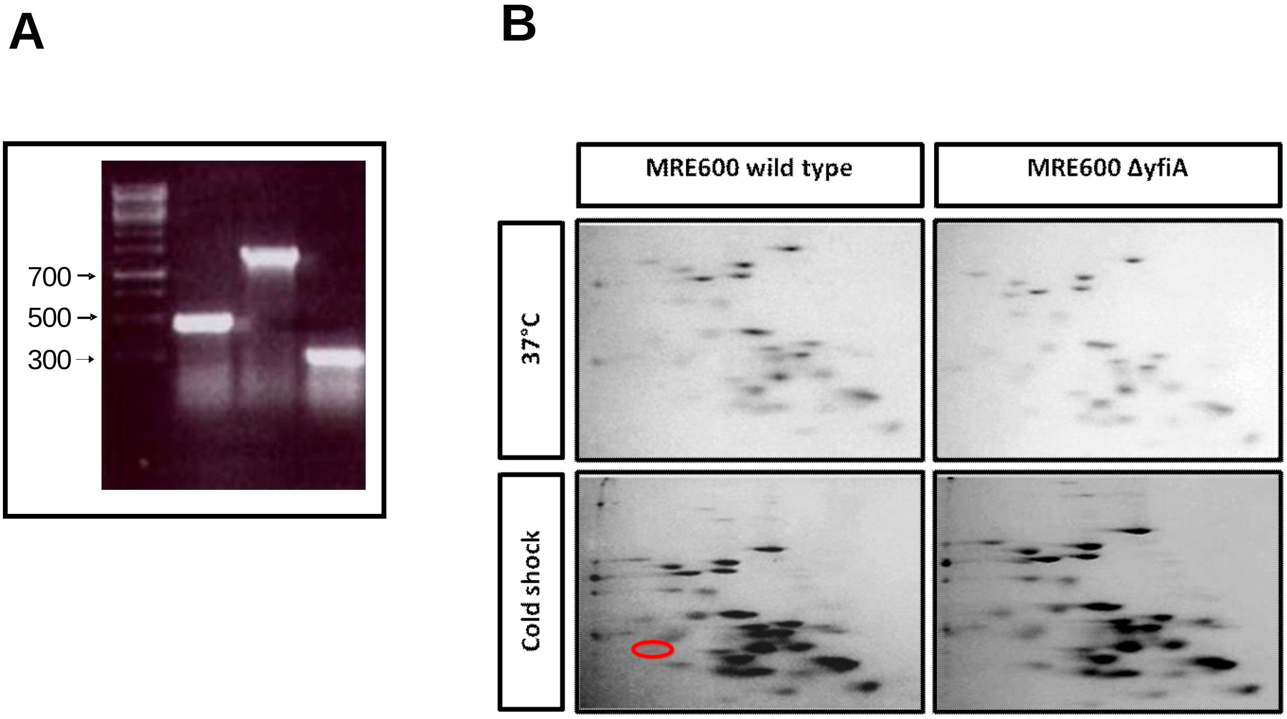


**Fig. S1.** Assessment of MRE600 *ΔyfiA* mutation. (A) PCR of *yfiA* locus. Lane 1: 100 bp DNA ladder (Fermentas); lane 2: amplification of the *E. coli* wt *yfiA* locus; lane 3: amplification of the *yfiA* locus after recombination with a kanamycin cassette (*yfiA::kan*); lane 4: amplification of the *yfiA* locus after kanamycin cassette excision (*ΔyfiA*). (B) 2D-gel electrophoresis analysis of 70S ribosomal proteins isolated from *E. coli* MRE600 wt (left) and *ΔyfiA* cells (right) grown at 37°C (upper panels) or subjected to 60 min cold-shock (lower panels). The spot corresponding to protein PY is circled.
Further details are given in Experimental procedures.

# PAGE 15%

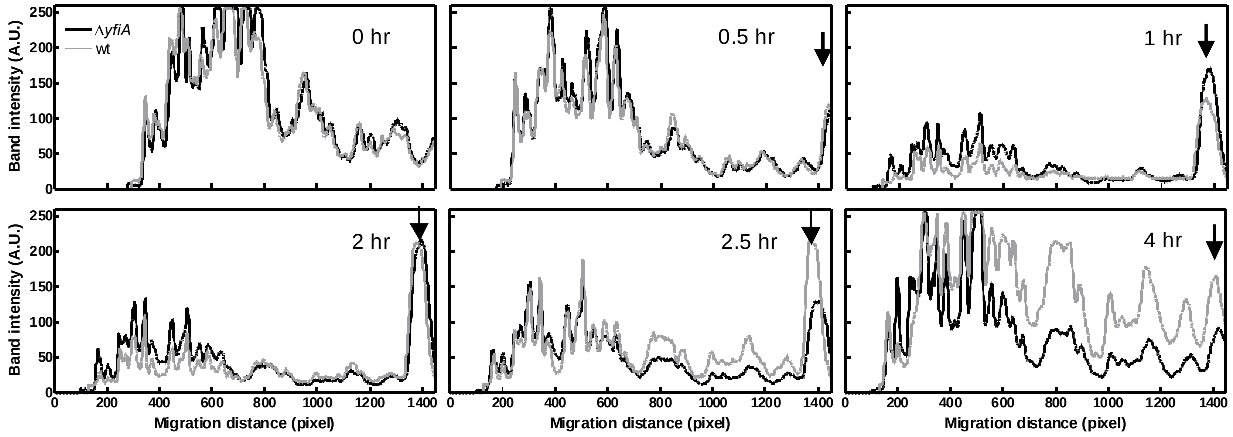


# PAGE 10%

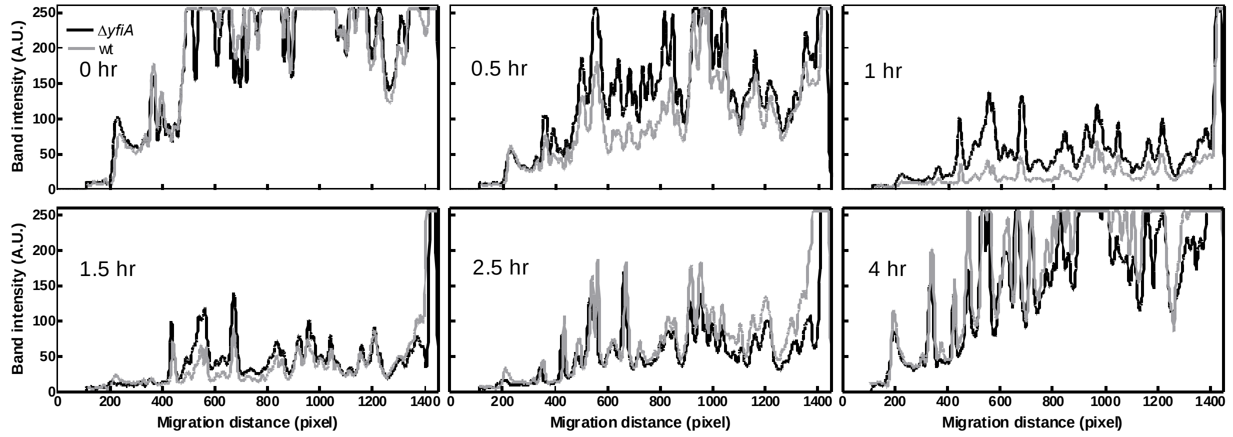


# PAGE 7%

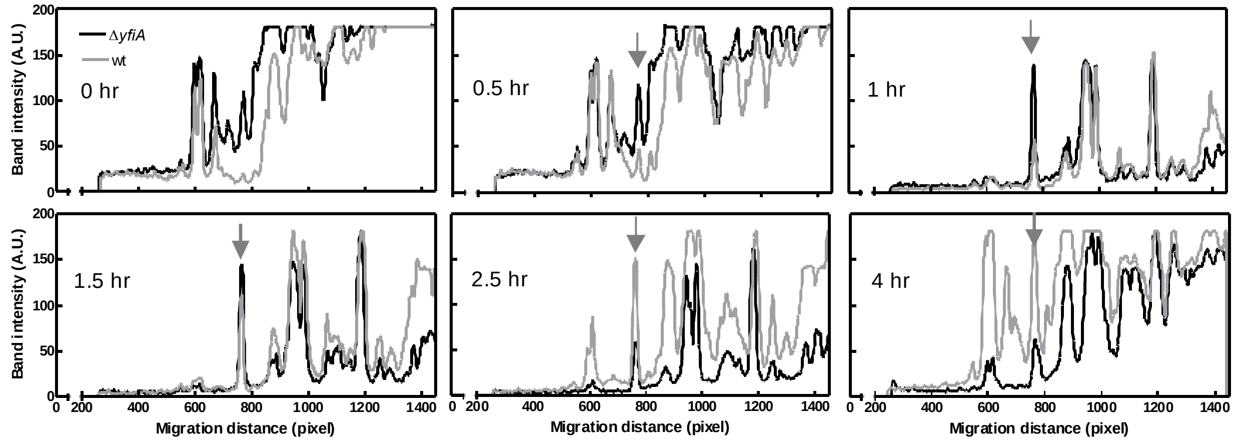

**Fig. S2.** *In vivo protein* expression after cold shock. Synthesis of the proteins in MRE600 wt (grey lines) or MRE600*ΔyfiA* (black lines) was followed by giving pulses of [$^{35}$S]-Pro-mix, as described in Experimental Procedures, immediately before (time 0) or at the times following cold shock indicated in the graphs. After chasing with an excess of unlabelled Met and Cys, the samples were processed for the electrophoretic separation at 7%, 10% and 15% acrylamide concentrations and for the determination of the radioactivity using a Molecular Imager (Bio-Rad GS 250).
The peaks in the graphs correspond to the intensity, expressed as “Arbitrary Units”, of the same point of each band present in the lanes of the various gels.
The black and gray arrows indicate the pick corresponding to CspA and another unidentified cold-shock protein, respectively.

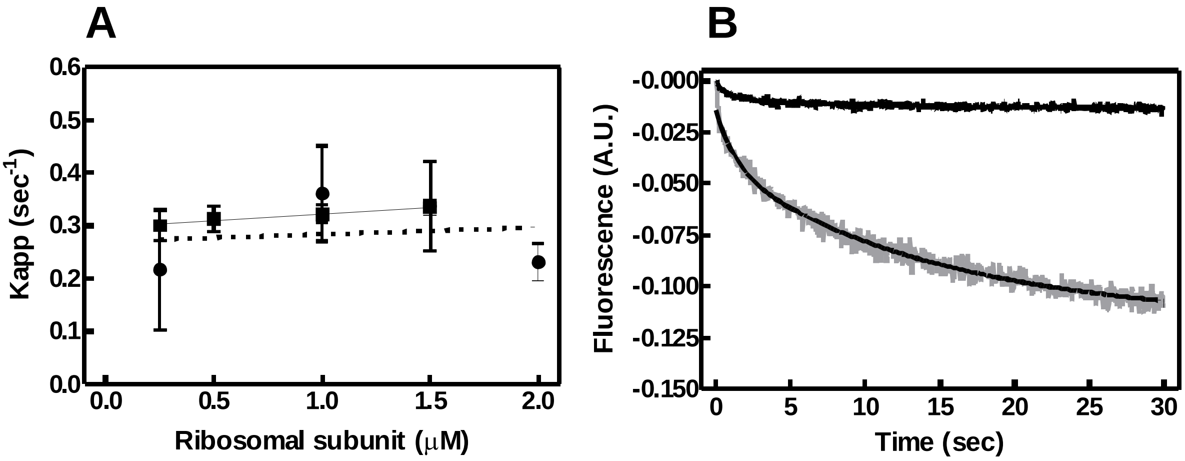


**Fig. S3.** A) Concentration dependence of $k_{app2}$ from the binding of PY to the 30S subunit (■) or the 50S subunit (●). The *y*-axis intercept gives the value of the backward rate constant $k_{off}$, while the slope of the lines corresponds to the forward rate constant $k_{on}$ of the reactions. Standard deviations were calculated from at least 10 different time courses.
B) Time courses of PY dissociation from 30S subunit (grey) or 50S subunit (black) monitored by PY_Alexa555 fluorescent change. 0.6 µM of PY_Alexa555 and 0.2 µM of 30S or 50S subunits were pre-incubated at 15°C and then rapidly mixed with the reaction buffer. Further details are given in the text and in Experimental Procedures.
